# Characteristics and limitations of a secondary dose check software for VMAT plan calculation

**DOI:** 10.1002/acm2.13206

**Published:** 2021-03-05

**Authors:** Andrew J. Shepard, Sean P. Frigo

**Affiliations:** ^1^ Department of Human Oncology School of Medicine and Public Health University of Wisconsin‐Madison Madison WI USA; ^2^ Present address: Department of Radiation Oncology University of Iowa Iowa City IA USA

**Keywords:** dosimetric leaf gap, leaf offset, MLC model, Mobius3D, secondary dose check

## Abstract

**Purpose:**

To assess the implementation, accuracy, and validity of the dosimetric leaf gap correction (DLGC) in Mobius3D VMAT plan calculations.

**Methods:**

The optimal Mobius3D DLGC was determined for both a TrueBeam with a Millennium multi‐leaf collimator and a TrueBeamSTx with a high‐definition multi‐leaf collimator. By analyzing a broad series of seven VMAT plans and comparing the calculated to the measured dose delivered to a cylindrical phantom, optimal DLGC values were determined by minimizing the dose difference for both the collection of all plans, as well as for each plan individually. The effects of plan removal from the optimization of the collective DLGC value, as well as plan‐specific DLGC values, were explored to determine the impact of plan suite design on the final DLGC determination.

**Results:**

Optimal collective DLGC values across all energies were between −0.71 and 0.89 mm for the TrueBeam, and between 0.35 and 1.85 mm for the TrueBeamSTx. The dose differences ranged between −6.1% and 2.6% across all plans when the optimal collective DLGC values were used. On a per‐plan basis, the plan‐specific optimal DLGC values ranged from −4.36 to 2.35 mm for the TrueBeam, and between −1.83 and 2.62 mm for the TrueBeamSTx. Comparing the plan‐specific optimal DLGC to the average absolute leaf position from the central axis for each plan, a negative correlation was observed.

**Conclusions:**

The optimal DLGC determination depends on the plans investigated, making it essential for users to utilize a suite of test plans that encompasses the full range of expected clinical plans when determining the optimal DLGC value. Validation of the secondary dose calculation should always be based on measurements, and not a comparison with the primary TPS. Varying disagreement with measurements across plans for a single DLGC value indicates potential limitations in the Mobius3D MLC model.

## 1 INTRODUCTION

Secondary dose calculations are utilized to serve as an independent verification of the primary treatment planning system (TPS). This independence is enhanced when the secondary check arrives in a pre‐configured state with a standard beam model for a given machine/energy class. In this case, it is recommended that the user makes minimal, if any, modifications to the software's model configuration. When changes are made, it is essential to have an understanding of the modifiable factors and their impact on dose calculations, as well as how parameter value variation relates to a measurement scenario.

Mobius3D (Varian Medical Systems Inc., Palo Alto, CA) is a secondary check software that provides quality assurance calculations for a full range of clinical photon and electron plans. For photon beams, Mobius3D utilizes a CT dataset along with plan and structure information (exported from the primary TPS) to perform an independent convolution‐superposition dose calculation for comparison with the dose calculated by the primary treatment planning system.^1^ Vendor‐specific beam models which contain a set of default values are provided. Outside of the necessary linac calibration conditions, it is recommended that the beam model is maintained as provided unless large variations are observed between the reference and measured percent depth dose (PDD) and off‐axis ratios (OAR).

Customization of the beam‐model may be facilitated with an auto‐modeling feature at the expense of reducing the independence of the beam model.^2^ In addition to built‐in parameters, there is a series of machine configuration parameters whose values the user can adjust, including those related to the determination of treatment times (dose rate), deliverability (max MU/field), clearance (distance between isocenter and collimator surface; collimator radius), and dose (dosimetric leaf gap correction). Of these parameters, the dosimetric leaf gap correction (DLGC) is the only one that will directly affect the dose calculation for MLC‐based plans, and subsequently the dose calculation accuracy of the system.

The Mobius DLG parameter contains two parts, an internal value (DLGI), which is not exposed to the user and is determined by automodeling routines executed by the vendor, and a correction (DLGC) which the user can vary. Prior to calculation, each MLC leaf position value in Mobius is moved by an amount of DLG/2 = (DLGI + DLGC)/2. There are specific DLGI and DLGC values for each machine and energy combination. According to Varian documentation, the DLGC in Mobius3D is modifiable by the user on a per‐machine, per‐energy basis to help account for differences between real‐world dosimetry and the inherent Mobius MLC transmission model.^2^ Making the DLGC more positive increases the calculated dose, while making it more negative decreases the calculated dose. Essentially, the Mobius3D DLG can be interpreted as a single entry to a leaf offset table within the software, where the DLGC is used to tune the internal DLGI to the institution's preference.

An important distinction when considering the Mobius3D DLG is that it is not defined in the same manner as the DLG in the Eclipse treatment planning system (DLG_E_). In Eclipse, the DLG_E_ is an inherent part of the step‐wise transmission function utilized to model the rounded leaf tip of the MLC. There is no consideration of leaf height and no leaf offset table. Alternatively, Mobius3D utilizes a full rounded leaf‐tip calculation using ray‐tracing and considering the leaf height to define the transmission function, which results in a smooth falloff of the fluence through the rounded leaf end.^3^

There have been investigations reported in the literature regarding the commissioning, validity and implementation of Mobius3D for use as a secondary dose calculation tool.^4^, ^5^, ^6^, ^7^, ^8^ On average, prior works saw typical agreement relative to ion chamber measurements in a phantom of <2%,^4^, ^6^, ^7^, ^8^ though differences as large as 5.5% were reported.^8^ These authors focused on the overall implementation of the software and validated the use of Mobius3D as a suitable secondary check software for treatment planning systems used within their clinic. Hillman et al. also investigated the Mobius3D MLC model as applied specifically to SRS/SBRT treatments and looked at a revised MLC model.^9^ Further, they investigated an alternative DLGC determination method that relied on sweeping gap plans, though noted that ultimately a DLGC optimization based on patient plans is likely necessary.^9^ Work by Kim et al. investigated the MLC modeling accuracy in Mobius3D, observing higher uncertainties in the dose calculation for small fields, and noting that careful optimization of the DLGC is necessary for optimal performance.^10^

This work presents in‐depth the determination and implications of the DLGC for a full range of clinically relevant plans, including both large‐field VMAT and small‐field SBRT cases. The optimization of the Mobius3D DLGC for both a TrueBeam with a Millennium MLC (MMLC) and a TrueBeamSTx with a high‐definition MLC (HDMLC) emphasizes the necessity for a representative set of test plans in DLGC optimization. The results show how a test plan suite in general plays in to TPS validation.

## 2 MATERIALS AND METHODS

### 2.A Collective DLGC optimization

The optimal DLGC value was determined for a TrueBeam with an MMLC and energies of flattened 6, 10, and 15 MV, as well as unflattened 6 and 10 MV. It was also determined for a TrueBeamSTx with an HDMLC and the same energies except 15 MV flattened. The DLGC was determined for each energy on each machine independently following an optimization process that was a variation of that recommended by Mobius3D,^2^ where our approach utilized multiple ion chamber measurements (in target, both on‐ and off‐axis). The optimal collective DLGC value for each energy was determined by comparing the Mobius3D calculated dose at several DLGC values to the measured ion chamber dose for a series of test plans.

Seven VMAT test plans were utilized throughout the optimization: four geometrically based, and three anatomically based. The four geometrically based plans were based on recommendations made in TG‐119 and contained either a central cylinder, lateral cylinder, large cylinder, or c‐shape target structure.^11^ The anatomically based plans consisted of a representative head and neck, lung SBRT, and chest wall targets. The plans were chosen to cover a range of the clinical scenarios expected at our institution. An overview of each plan and the corresponding target structure is provided in Table 1.

**TABLE 1:** Target structure details. Relevant volume and positional statistics for the target structure in each plan used for DLGC optimization.

Plan	Target volume [cm^3^]	Absolute distance from isocenter [cm]
Central cylinder	221.5	0.01
C‐Shape	274.2	0.26
Large cylinder	1616.2	0.02
Lateral cylinder	221.5	10.00
Neck	329.3	3.63
Lung SBRT	15.5	0.21
Chest wall	1634.2	4.13

**FIG. 1:**
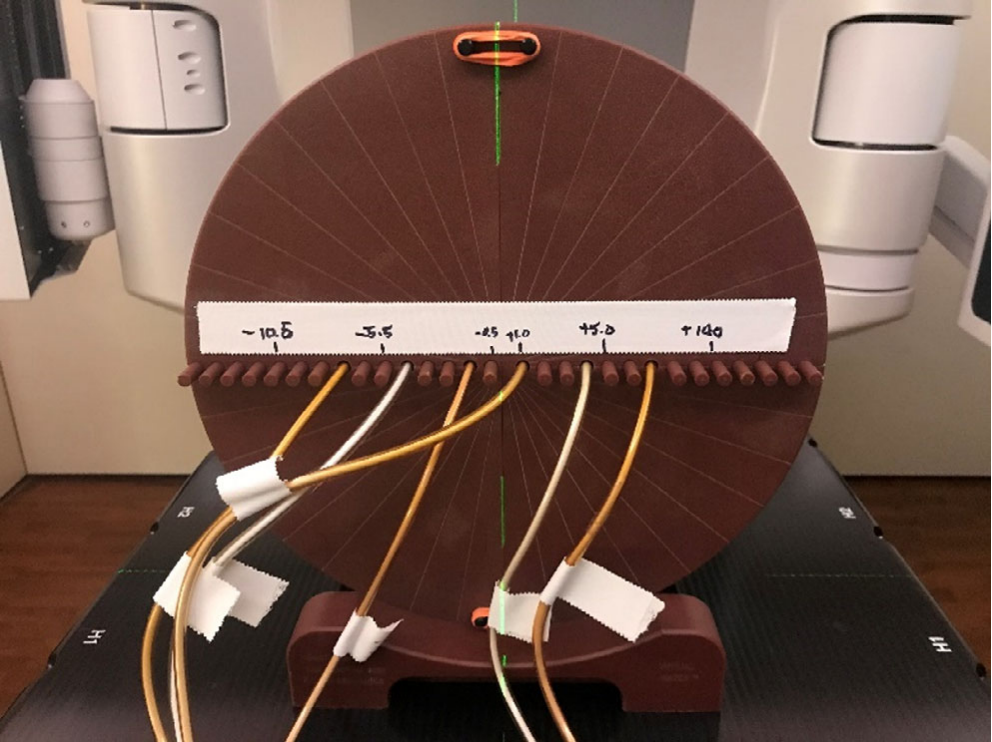
Tomotherapy “cheese” phantom measurement setup. The phantom was positioned with the ion chamber inserts located laterally across the central slice. Ion chambers were adjusted on a per‐plan basis such that they were in the target location for each plan.

All plans were delivered to a cylindrical TomoTherapy “cheese” phantom (diameter = 30 cm, length = 18 cm; Accuray Inc., Sunnyvale, CA), which allowed for the placement of six A1SL ion chambers (r_cav_ = 2.00 mm; collecting volume = 0.053 cm^3^; Standard Imaging Inc., Middleton, WI) laterally along the central slice of the phantom. The ion chambers were positioned such that measurements were acquired at representative target (high‐dose) locations for each of the plans investigated. An example of the setup used for measurement acquisition is shown in Fig. 1. Our configuration differed slightly from the recommendation in Mobius3D documentation for the determination of the DLGC, which recommended the use of the Mobius Verification Phantom and a single cylindrical ion chamber.^2^ The decision to use the cheese phantom with multiple A1SL ion chambers was made based on the availability of equipment in our clinic, the desire to acquire multiple measurements for each plan at a variety of positions, and the utilization of smaller ion chamber measurement volumes.

For each plan, the ion chambers were placed in high‐level, low‐gradient dose regions corresponding to the treatment target volume. The charge was measured and converted to absolute dose using appropriate chamber correction and calibration factors. Absolute dose uncertainty for the A1SL was estimated to be 1%.^12^ The percent difference between the calculated and measured dose was calculated for each chamber location, defined as

$$\text{Percent Difference}=100\times \frac{\left(Calculated‐Measured\right)}{\mathrm{Measured}}$$

This formulation considers the measurement as the reference and produces a result that gives a direct indication of whether the calculation is higher or lower than the measurement. Chambers were only considered if they had a measured dose of >80% of the maximum calculated dose.

**FIG. 2:**
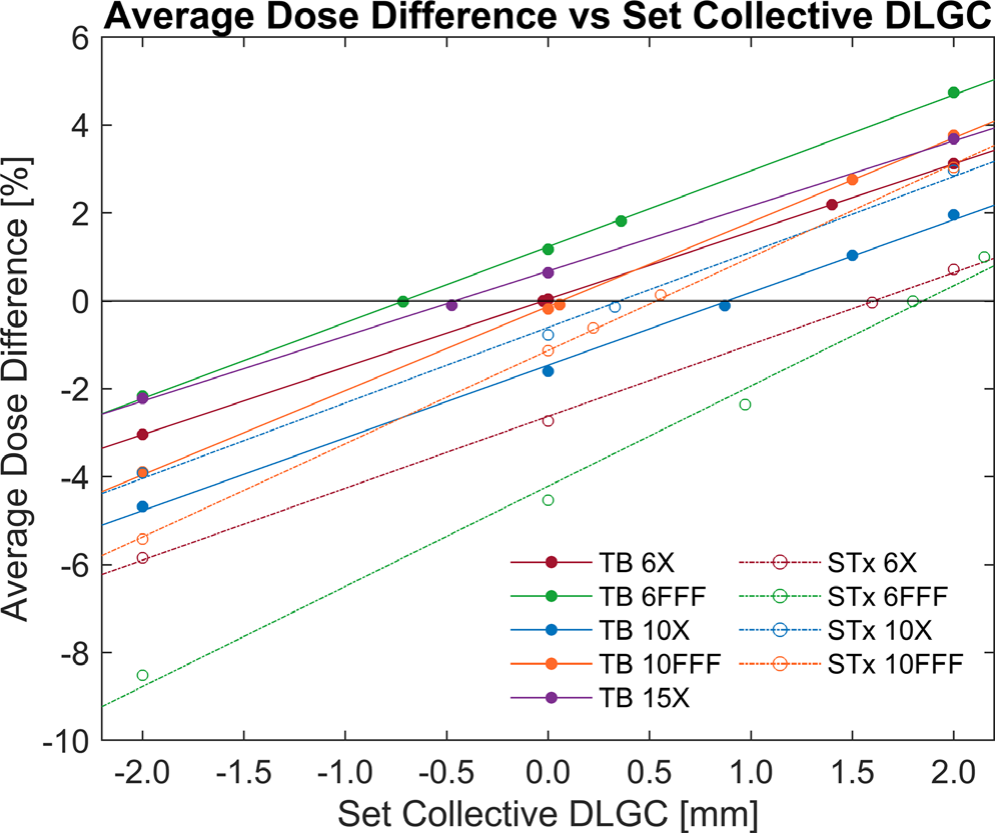
Average Dose Difference vs. Collective DLGC. The average dose difference relative to measurements across all seven test plans for a given collective DLGC, machine, and energy. The optimal collective DLGC for each machine/energy was determined by fitting the data linearly and identifying the value for 0.0% absolute average dose difference. Each data point represents the percent difference averaged across the seven test plans.

For each machine/energy/MLC/plan combination, Mobius3D (v2.1) calculations were performed for DLGC values between −2.00 and 2.15 mm, in variable steps as shown in Fig. 2, and were then compared to measurements by calculating the percent difference. The explored DLGC values investigated were chosen to ensure that the optimal collective DLGC value would be contained within the range of these values, and interpolation to an optimal result would be possible.

To facilitate dose comparisons, ROI structures representative of the collection volumes of the ion chambers were defined in the RayStation (v7.0.0.19) treatment planning system (RaySearch, Stockholm, Sweden) and exported to Mobius3D. The average dose difference across eligible chambers was first calculated for each plan, followed by an average across all plans to determine the total average percentage dose difference at a given DLGC value. A linear fit was applied to the total average dose difference (all plans and eligible chambers for a given machine/energy) for either four or five DLGC exploration values, and based on the fit, the collective DLGC corresponding to the minimum absolute dose difference was taken to be the optimal value for the specific energy and machine.

### 2.B Individual plan DLGC optimization

In addition to determining the optimal collective DLGC, the optimal DLGC for each individual plan was also calculated in an identical manner, as an investigation into the variability of the DLGC based on plan characteristics. As opposed to the collective DLGC, the plan‐specific values will be referred to in this work as the plan‐specific DLGC.

The plan‐specific values were calculated for each machine and energy independently based on the line of best‐fit for each individual plan. It was observed that some of the optimal plan‐specific DLGC values fell outside of the initial values set for the collective DLGC optimization, so spot‐check calculations were performed for those plans which required extrapolation. This was in order to verify the validity of the best‐fit curve. In the scenario that the extrapolated value still resulted in a dose difference of >1% (estimate of absolute dose determination uncertainty for A1SL^12^), the new data point was added to the analysis, and a new best‐fit for that specific plan was determined.

The importance of each plan to the determination of the collective DLGC was assessed by performing a collective DLGC optimization while excluding single plans from the analysis, in order to assess the potential impact of test plan selection.

### 2.C Plan‐specific DLGC correlation to plan complexity metrics

The optimal values for the plan‐specific DLGC were assessed relative to several plan metrics to assess whether any relevant correlation between DLGC values and plan metrics was present. These metrics were calculated based on work by Desai et al. and included the average absolute leaf position off the central axis (ALPCA), mean aperture displacement (MAD), and modulation complexity score (MCS), among others.^13^

### 2.D Comparison with RayStation calculations

To investigate how the dose differences observed in Mobius3D compared with the primary TPS at our institution, all seven plans were initially calculated in RayStation using standard clinical planning procedures, and then exported to Mobius3D. The calculated average dose to relevant A1SL ion chamber structures in RayStation was compared to the corresponding average values to the same structures in Mobius3D. The differences were analyzed for their range and average value for a given plan/energy combination. We then observed the level of agreement between the primary TPS and the secondary check software relative to measurements for the test plans, to help aid in the determination of relevant action criteria when comparing the secondary check software to the primary TPS.

## 3 RESULTS

### 3.A Collective DLGC optimization

The average dose difference between the calculated and measured dose across all plans investigated is presented in Fig. 2 for a range of explored DLGC values for each energy and machine. The linear fits had an average R^2^ of 0.998. The optimal collective DLGC, the value at which the fit equaled an average dose difference of 0.0%, for all machines and energies is presented in Table 2.

**TABLE 2:** Optimal collective DLGC. The optimal collective DLGC value for each energy and machine.

Optimal collective DLGC [mm]					
	6X	10X	15X	6FFF	10FFF
TrueBeam	−0.02	0.89	−0.46	−0.71	0.07
TrueBeamSTx	1.61	0.35	–	1.85	0.53

**FIG. 3:**
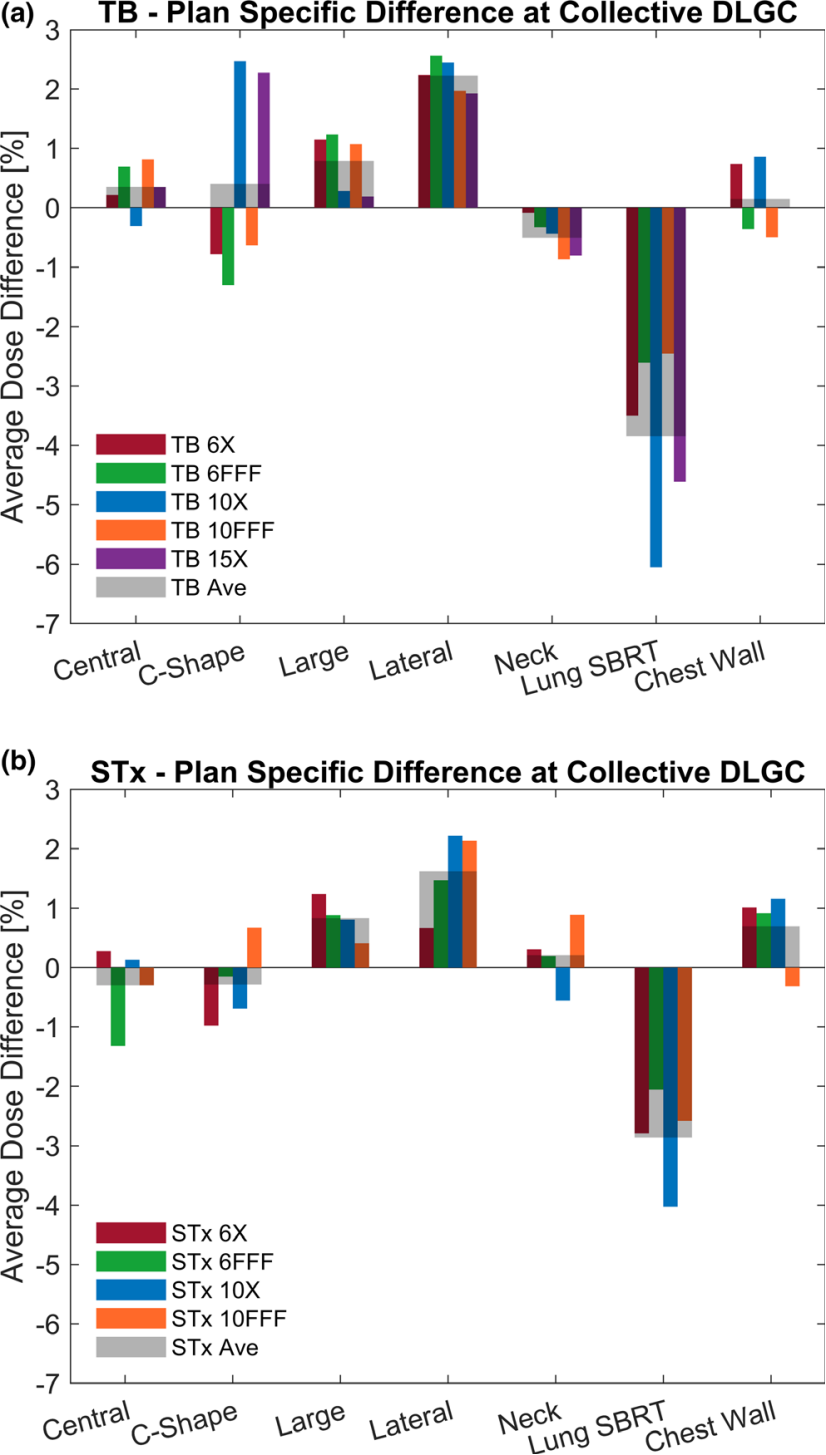
Plan Specific Dose Deviation at Optimal Collective DLGC. The dose deviation between Mobius3D calculated dose and measurement at the optimal collective DLGC for each plan and energy on the (a) TrueBeam with MMLC and (b) TrueBeamSTx with HDMLC is presented. Measurements for the lateral cylinder plan was noted to be consistently lower than calculations, while measurements for the lung SBRT plan was consistently higher than calculations; others show mixed agreement.

Although the optimal collective DLGC corresponded to the value at which the average dose difference of all plans was equal to zero, there were variations in the dose difference within a given machine and energy when the optimal collective value was used. This is shown in Figs. 3(a) and 3(b), which present the average dose difference for each plan using the optimal collective DLGC for each energy on the TrueBeam and TrueBeamSTx, respectively. Additionally, these figures display the average dose difference across all beam energies for each plan investigated.

It was observed that at the optimal collective DLGC, the average dose difference relative to measurements (±1 standard deviation) when considering plan and energy independently was −0.1% ± 2.0% (range: −6.1% to 2.6%) for the TrueBeam, and −0.0% ± 1.5% (range: −4.0% to 2.2%) for the TrueBeamSTx. Considering only the magnitude of the dose difference relative to measurements, the average across all energies and plans investigated was 1.4% ± 1.4% (range: 0.0%–6.1%) for the TrueBeam, and 1.1% ± 0.9% (range: 0.1% to 4.0%) for the TrueBeamSTx. At the plan level, the most significant dose differences occurred for the lateral cylinder and lung SBRT plans. Mobius3D consistently calculated doses greater than measurements for the lateral cylinder plan, and less than measurements for the lung SBRT plan. The average magnitude of the dose difference across both machines was 2.0% ± 0.6% for the lateral cylinder plan, and −3.4% ± 1.3% for the lung SBRT plan.

### 3.B Individual plan DLGC optimization

**FIG. 4:**
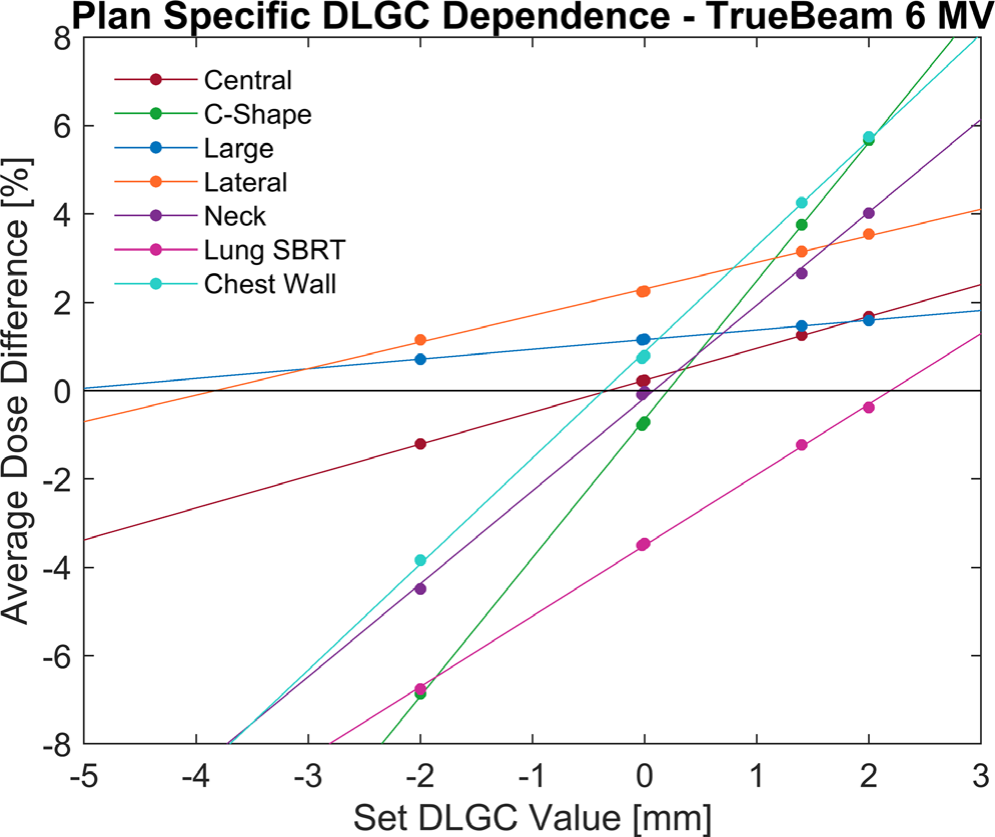
Plan‐Specific DLGC Analysis. The dose difference between the calculated and measured dose at varying DLGC values for each plan as calculated for the 6 MV flattened beam model on the TrueBeam. The points indicate explicit calculations that were performed, while the solid line indicates the line of best fit for each plan.

**FIG. 5:**
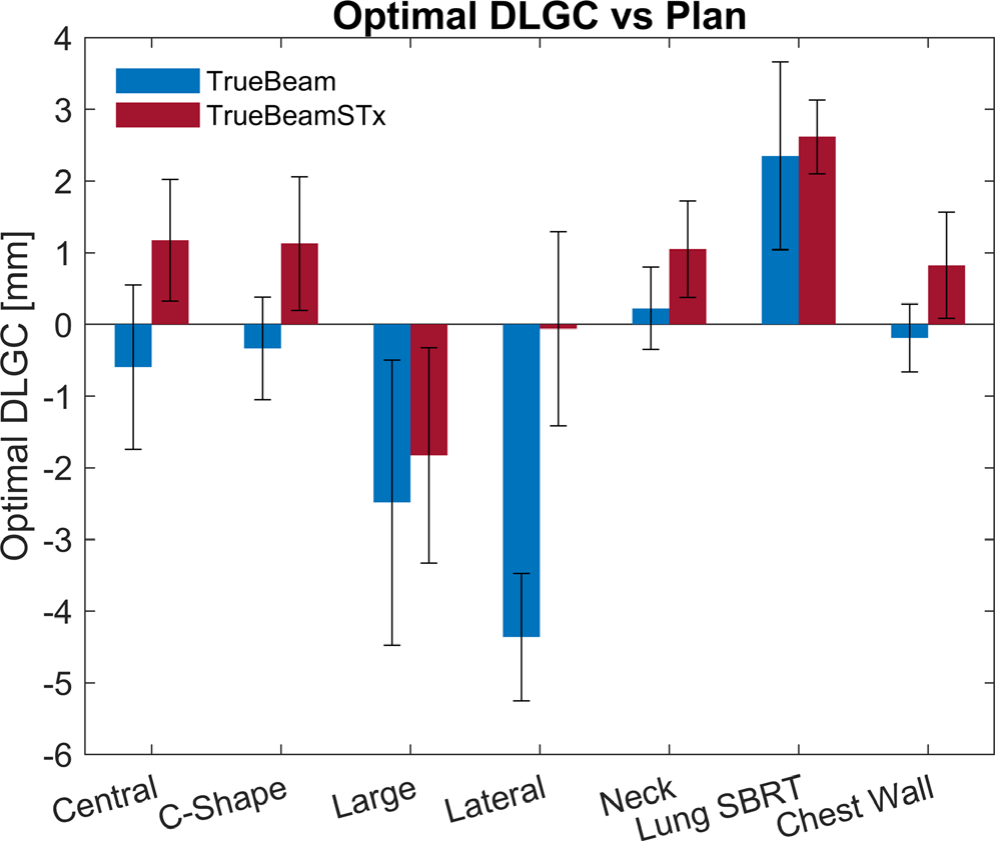
Optimal DLGC vs. Plan. The optimal plan‐specific DLGC across all energies for a given machine is presented for each plan. The error bars represent the standard deviation of the dose difference for all energies for a given plan.

Due to the dose differences observed for individual plans at the optimal collective DLGC, an identical DLGC analysis was performed on a per‐plan basis. This calculation was performed for each machine and energy combination, and an example of the analysis for the 6 MV beam on the TrueBeam model is provided in Fig. 4. All extrapolated values were spot‐checked, and the percent difference was within ±0.6% at the extrapolated DLGC value. Considering each plan and energy individually resulted in DLGC values between −5.38 and 4.59 mm. The optimal plan‐specific DLGC averaged over all energies is shown in Fig. 5, with values ranging from −4.36 to 2.62 mm. By presenting these data as an average across all energies, it helps isolate the plan‐specific variability in the values observed.

### 3.C Plan‐specific DLGC correlation to plan complexity metrics

**FIG. 6:**
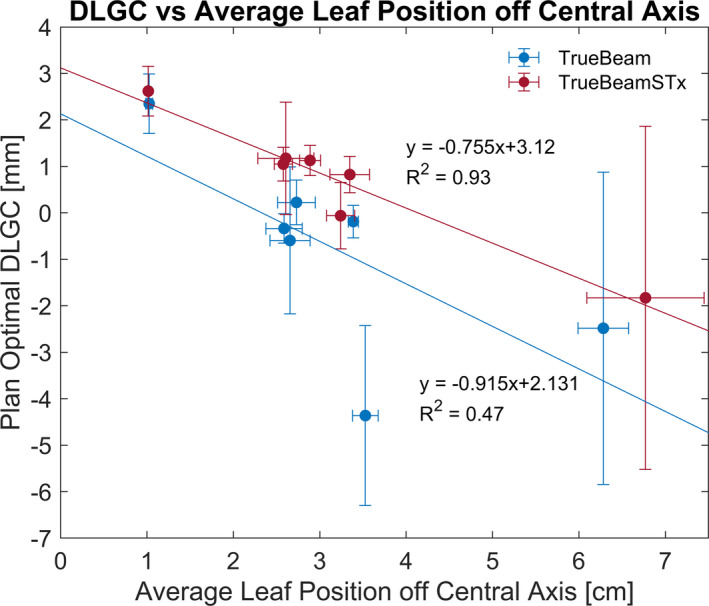
Optimal DLGC vs. Average Leaf Position off Central Axis. The optimal DLGC averaged across all energies is compared to the MLC positioning characteristics of the plan. As the average leaf position off the central became larger, the optimal DLGC became more negative. DLGC error bars are representative of a 1% dosimetric uncertainty and ALPCA error bars are given by the standard deviation across all energies.

To assess the potential dependence of the DLGC on plan characteristics and MLC position distribution, the average absolute leaf positions from the central axis (ALPCA), mean aperture displacement (MAD), and modulation complexity score (MCS) were calculated for each plan. The optimal plan‐specific DLGC value averaged over all energies was compared to each plan metric. The Pearson correlation coefficient was −0.69, −0.00, and −0.02 for the ALPCA, MAD, and MCS, for the TrueBeam, and −0.97, −0.33, and −0.39 for the TrueBeamSTx. Low correlation values were observed between the plan‐specific DLGC values and both the MAD and MCS metrics; however, there was a relatively high correlation observed with the ALPCA. Figure 6 shows the energy‐averaged plan‐specific DLGC values compared to the ALPCA, along with a linear best‐fit. Plans with a smaller leaf position from the central axis typically required a more positive DLGC, demonstrating a negative linear trend. To demonstrate the sensitivity associated with each plan to varying DLGC values, the error bars presented in Fig. 6 are representative of a ±1% uncertainty in the dose measurement. Note that plans with a lower DLGC sensitivity, as demonstrated by a lower slope in Fig. 4, correspondingly had a larger optimal DLGC uncertainty for a constant ±1% dose uncertainty.

### 3.D Effects of plan exclusion from optimization

To assess the potential impact that plan selection can have on the optimal collective DLGC determination, the same analysis was repeated after removing a single plan and the change in the collective DLGC was noted. This was performed independently for an exclusion of both the lung SBRT plan and the lateral cylinder plan (representative of the plans exhibiting the largest dose differences when the optimal collective DLGC value was used).

**FIG. 7:**
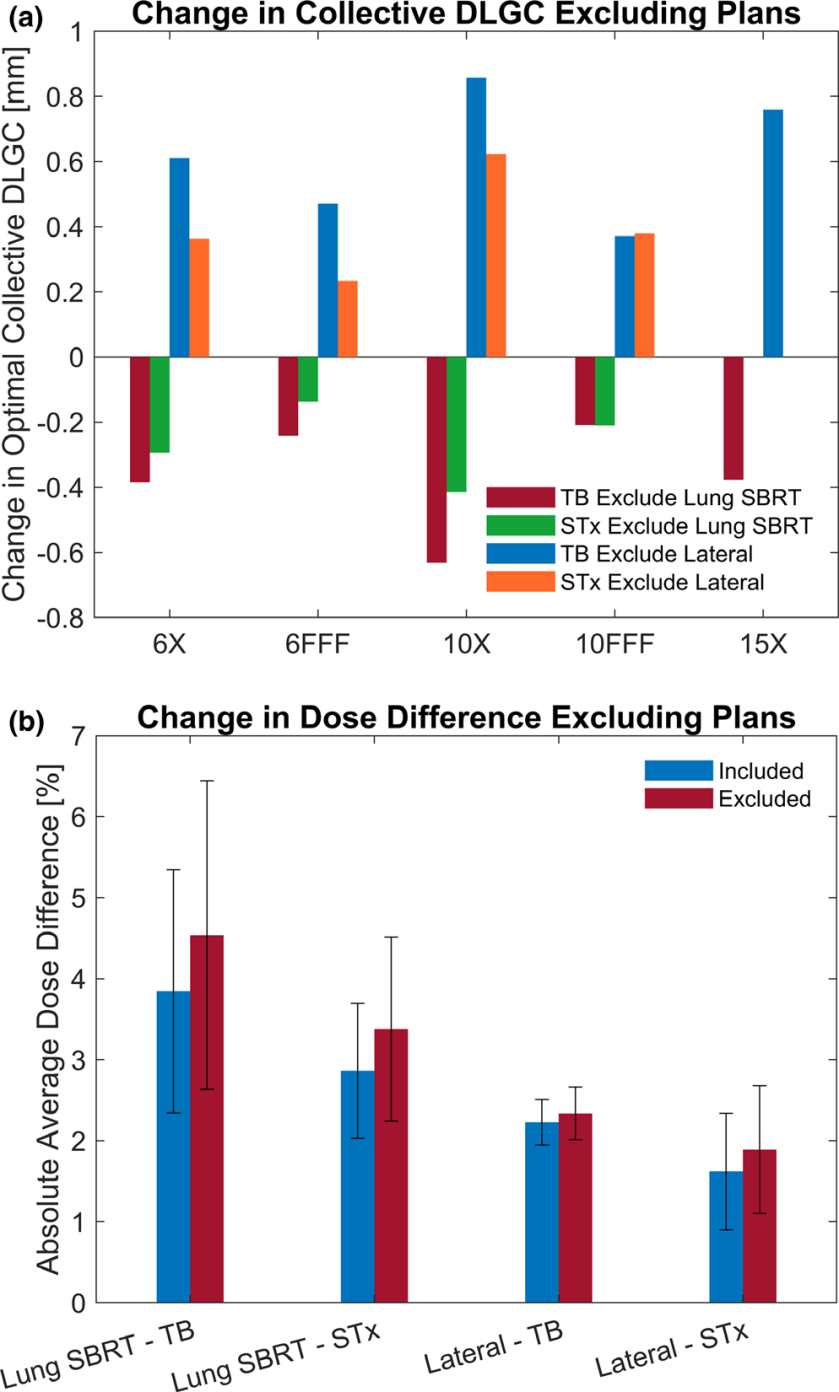
Effects of Plan Exclusion. The change in the optimal DLGC while excluding the lung SBRT or lateral cylinder plan as compared to the analysis including all plans is provided in (a). The dosimetric effect of applying the DLGC determined while excluding the given plan from the analysis is shown in (b).

With plan removal, the change in the optimal collective DLGC value ranged from −0.63 to 0.86 mm. The results for all energies are presented in Fig. 7(a). The removal of the lung SBRT plan from the analysis always resulted in a decrease in the collective DLGC, while the removal of the lateral cylinder plan always resulted in an increase in the collective DLGC. Further, applying the plan‐removal collective DLGC value to the two respective plans resulted in increases in the absolute dose differences of 0.6 ± 0.4% and 0.2 ± 0.1% for the lung SBRT plan and the lateral cylinder plan, respectively. These changes in the absolute dose difference observed with the plans excluded from the collective DLGC determination is demonstrated in Fig. 7(b).

### 3.E Comparison with RayStation calculations

Dose calculations were also performed with the RayStation treatment planning system for comparison with Mobius3D. On average, the difference between RayStation calculations and measurements, when considering each plan and energy independently, was −0.3 ± 0.9% (range: −2.4% to 1.3%) for the TrueBeam system, and 1.1 ± 0.8% (range: −0.4% to 2.6%) for the TrueBeamSTx system. The average magnitude of the dose difference of the RayStation calculations compared to measurements was 0.7 ± 0.6% (range: 0.0%–2.4%) on the TrueBeam system, and 1.1 ± 0.8% (range: 0.1%–2.6%) on the TrueBeamSTx.

Comparing to Mobius3D using optimized collective DLGC values, the magnitude of the calculated dose difference relative to measurements was less for RayStation in 24/35 and 12/28 plans for the TrueBeam and TrueBeamSTx machines, respectively. For RayStation and Mobius3D calculations relative to measurements for each plan, it was observed that the RayStation calculations agreed with measurements much better for the lung SBRT plan. The average dose difference magnitude across all energies on both machines was 1.0 ± 0.7% (range: 0.1%–2.4%), whereas the Mobius3D average dose difference magnitude was 3.4 ± 1.3% (range: 2.1%–6.1%). The plan that Mobius3D outperformed the RayStation model to the largest extent was the chest wall plan, with an average absolute difference of 0.7 ± 0.4% (range: 0.0%–1.2%), compared to 1.2 ± 1.0% (range: 0.0%–2.6%) for the RayStation model.

Evaluating directly the Mobius3D calculations at the optimal collective DLGC relative to the RayStation calculations, the average dose difference was 0.2% ± 1.9% (range: −4.8% to 4.3%) for the TrueBeam, and −1.1% ± 1.6% (range: −5.2% to 1.7%) for the TrueBeamSTx. The largest dose differences between the two calculations were present for the lung SBRT plan where Mobius3D consistently calculated a dose lower than RayStation, with an average difference of −3.3% ± 1.1% (range: −5.2% to −2.2%).

## 4 DISCUSSION

Performing the DLGC optimization based on minimizing the calculated dose difference with measurements for seven VMAT plans allowed for determination of a single collective value for each energy and machine. As expected, the average dose difference followed a linear trend with DLGC. This produced an optimal collective DLGC value that results in a minimum dose difference of −0.1% ± 2.0% for the TrueBeam, and −0.0% ± 1.5% for the TrueBeamSTx. Based on the way in which the optimization was performed, values very near 0.0% were expected. They generally are in agreement with many of the prior publications addressing Mobius3D implementation.^4^, ^5^, ^6^, ^7^, ^8^ However, in the determination of the collective DLGC, a relatively large range of dose difference values about the mean was observed, as well as patterns that were similar across all energies and machine/MLC models, but varying by plan. This study observed disagreement between Mobius3D and measurements of up to 6.1% for the TrueBeam and 4.0% for the TrueBeamSTx when the collective DLGC parameter value was used. Fontenot saw disagreement as large as 5.5%,^8^ but most prior publications reported values closer to 2–3%.^4^, ^5^, ^7^

Mobius3D is often used with relatively large gamma parameter tolerances of 5%/3 mm, providing an effective means for detecting gross errors and differences with the treatment planning system. As clinics look to potentially implement tighter action criteria, it is essential that they do so keeping current limitations in mind. If the goal is to focus the scope on a small number of plans, and the DLGC has been optimized specifically for the given plan characteristics, it may be reasonable to decrease the tolerance values, as in general, relatively good agreement between Mobius3D and measurements was observed. However, when considering a wide range of potential plans as was investigated in this work, a tightening of the action criteria could unduly raise flags due to plan disagreement from calculation inaccuracy. In this scenario, it may be difficult to truly determine whether disagreement between Mobius3D and the primary calculation is due to a problem with the plan, the primary TPS, or inaccuracies in the Mobius3D dose calculation. This was effectively demonstrated when comparing Mobius3D to RayStation calculations for the lung SBRT plan. Dose differences in the calculation of the lung SBRT plan by RayStation and Mobius3D on the order of 2%–5% indicate a tightening of the action criteria could result in the onset of false positives. In a similar comparison of RayStation and Mobius3D, Kim et al. observed large discrepancies for small‐field cases, which would be characteristic of the lung SBRT case investigated in this work.^10^

The large maximum differences observed in the current study are believed to potentially be due to the variability in plan characteristics investigated and the possibility that a single collective DLGC value is not adequate. To further investigate the potential variability in the DLGC with varying plan characteristics, an analysis was performed on a per‐plan basis. By optimizing the DLGC for each plan, energy, and machine, the optimal plan‐specific DLGC values ranged between −5.38 and 4.59 mm. Some of the plans had a shallower slope in the dose difference versus DLGC data, indicating that a wide range of DLGC values may result in a minimal dose difference for these types of plans. However, the overall observed variations in the optimal plan‐specific DLGC indicates how the choice of plans used for the commissioning process can have a large impact on the collective DLGC that is ultimately chosen by the user, especially for plans whose slope (sensitivity) is higher.

Using plans that are representative of the full range of expected clinical plans is essential to the proper selection of the collective DLGC parameter value to be used. For instance, Fig. 7(a) shows that when the lung SBRT plan was removed from the optimization, the collective DLGC was reduced by 0.34 ± 0.17 mm. On the contrary, if the lateral cylinder were removed, the collective DLGC became more positive, increasing by 0.52 ± 0.21 mm. The ultimate effect of the variability that may result if a non‐representative suite of test plans was chosen for collective DLGC determination is demonstrated in Fig. 7(b). The decision to potentially exclude a given plan from the collective DLGC determination therefore can lead to significant changes in the resultantly determined optimal DLGC value. This is particularly true when considering cases that may define the limits of treatment, such as very small or large fields and is demonstrated by the lung SBRT case. Removing it from the collective DLGC determination for the TrueBeam resulted in the average dose difference increasing from 3.9% to 4.5%. Prior studies by Kim et al. as well as Hillman et al. noted inaccuracies in Mobius3D calculations for small‐field deliveries, further strengthening the assertion that it is necessary to ensure the DLGC is optimized for the relevant clinical plans.^9^, ^10^

In addition to target volume, the MLC position characteristics in the DICOM RT‐Plan data were used to further characterize the test plans, focusing on the average leaf position relative to the central axis. Essentially, plans with leaf positions further from the central axis required a more negative plan‐specific DLGC. With only the ability to define a single DLGC, the model may struggle to accurately calculate dose for plans with characteristics different than what was used for the determination of the collective DLGC. This point is illustrated in Fig. 6 where the optimal plan‐specific DLGC for a given plan (averaged across all energies) is plotted relative to the ALPCA through the course of the plan. Considering the results observed for plans with varying characteristics, multiple DLGC values may be necessary to ensure accurate calculation for all clinical scenarios. In the current framework of the software, this would require creating a separate machine for plans with different characteristics. Without the ability for the DLGC to easily vary with plan characteristics, the accuracy of the software may be limited at the boundary extents of the plan characteristic considered, as was the case for the lung SBRT plan.

This work focused on the implementation of the DLGC for VMAT deliveries; however, it is important to consider the potential impact on conformal arc deliveries as well. This is particularly relevant at target edges and for small‐field conformal arc plans where the DLG will constitute a larger fraction of the full irradiation field width. Users should be cautious in the implementation of large positive or negative DLGC values optimized based on larger fields and which could have a substantial impact on small‐field conformal arcs. It would be prudent of the user to verify the validity of DLGC values on a series of conformal arc test plans as well, if the desire is to implement the same model as was developed for the VMAT plans. This point further emphasizes the necessity to use, or at the very least validate, a full representative suite of test plans for the determination of the proper DLGC.

## 5 CONCLUSION

A Mobius3D DLGC optimization procedure was implemented on both a TrueBeam with an MMLC and a TrueBeamSTx with an HDMLC. Optimal DLGC parameter values based on all test plans investigated were able to be determined through the comparisons of calculated and measured dose. While the DLGC was able to be optimized for the collective dose difference of the plans, the optimal collective DLGC was not the same as the optimal DLGC for each plan individually. Ultimately, the DLGC value determined from the optimization procedure will be dependent on the test plan spectrum, making it essential for users to consider how the software will be used and the shortcomings that may result from a given test plan suite. In order to achieve an increased accuracy across a full spectrum of plans, it would likely be necessary for the MLC model to be improved. Based on these results, MLC model limitations in the second check software could have the possibility of raising a false positive. Lastly, a comparison between the primary TPS, secondary TPS, and measurement, points out that one must validate their secondary TPS against measurement, and not use agreement with the primary TPS for this purpose.

## CONFLICT OF INTEREST

The authors have no conflict of interest to report.
